# Supplementary material for: Japanese Encephalitis Enzootic and Epidemic Risks across Australia
Source: Viruses. 2023 Feb 6;15(2):450. doi: 10.3390/v15020450 (PMC9962251; doi:10.3390/v15020450)
Supplement: Supplementary file 1 [file viruses-15-00450-s001.zip › viruses-2141464-supplementary.pdf]

Supplementary Table S1. List of waterbirds suspected to be reservoirs, for whom ecological niche models were combined.

S/N	**Common name**	**Scientific name**	**Reference**
1	Intermediate egret	*Ardea intermedia*	Graham et al., (2019); Slater, P., Slater, P., & Slater, R. (2003).
2	Cattle egret	*Ardea ibis*	Graham et al 2019; Slater, P., Slater, P., & Slater, R. (2003).
3	Little egret	*Egretta garzetta*	Graham et al 2019; Slater, P., Slater, P., & Slater, R. (2003).
4	Eastern reef egret	*Egretta sacra*	Graham et al 2019, McKilligan, N. E. I. L. (2002); Slater, P., Slater, P., & Slater, R. (2003).
5	Eastern great egret	*Ardea modesta*	Graham et al 2019; Slater, P., Slater, P., & Slater, R. (2003).
6	Nankeen night-heron	*Nycticorax caledonicus*	Graham et al 2019, Maddock, 2011; Slater, P., Slater, P., & Slater, R. (2003).
7	White-faced heron	*Egretta novaehollandiae*	Graham et al 2019; Chambers and Lyon 2006; Slater, P., Slater, P., & Slater, R. (2003).
8	White-necked heron	*Ardea pacifica*	Graham et al 2019; Slater, P., Slater, P., & Slater, R. (2003).
9	Striated heron	*Butorides striatus*	Graham et al 2019; Slater, P., Slater, P., & Slater, R. (2003).
10	Black bittern	*Ixobrychus flavicollis*	Graham et al 2019; Slater, P., Slater, P., & Slater, R. (2003).
11	Australian bittern	*Botaurus poiciloptilus*	Graham et al 2019; Slater, P., Slater, P., & Slater, R. (2003).
12	Black-necked stork	*Ephippiorhynchus asiaticus*	Graham et al 2019; Clancy, G. P., & Ford, H. A. (2013); Slater, P., Slater, P., & Slater, R. (2003).

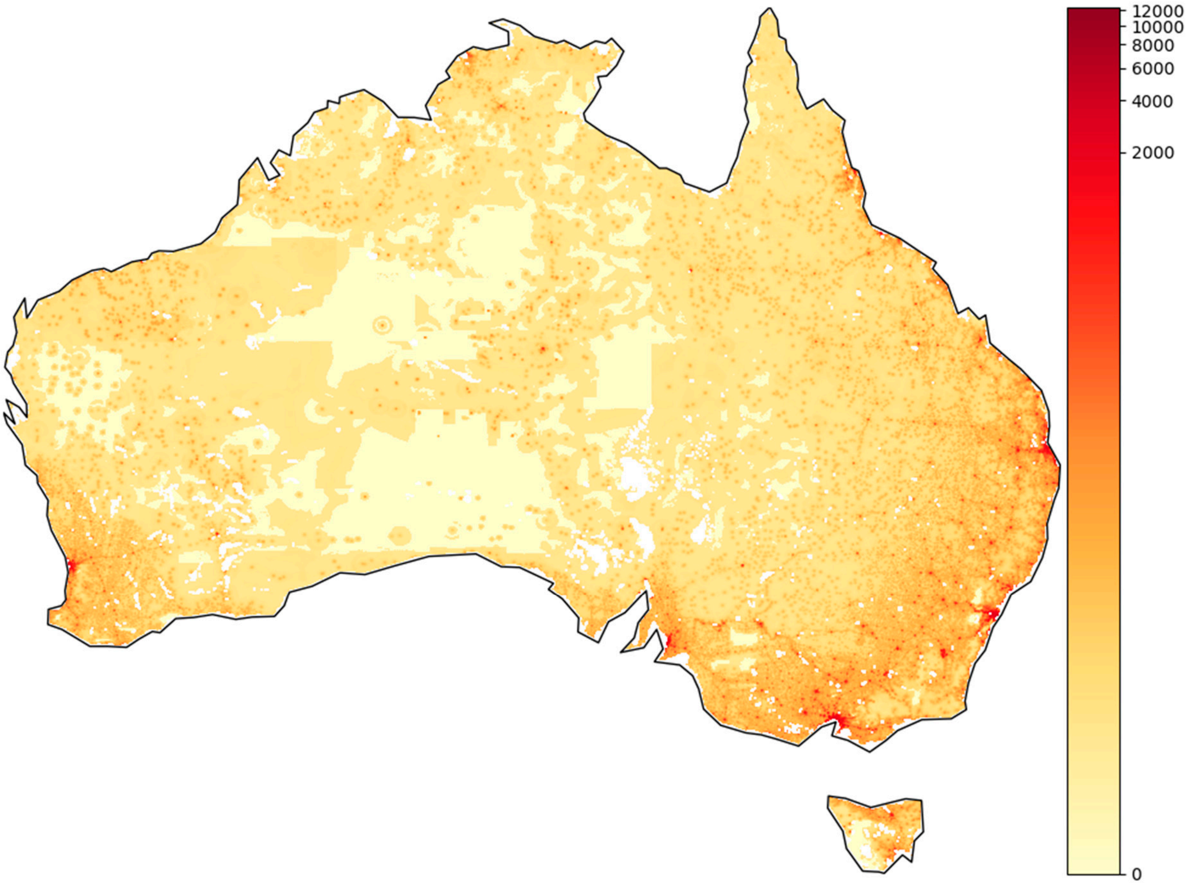

Supplementary Figure S1. Relative human population density across Australia, with the colour bar using a power law to enable visualisation of the distribution detail outside of the major cities (see Section 2.5 in the paper).

Supplementary File S1. Statistical approaches used in the study.

The probabilities of vector presence and wild bird presence were combined using the standard probabilistic approach,

$$k_s = \mathbf{1} - \left[\prod_{i=\mathbf{1}}^{\boldsymbol{N}} 1 - p_{i,s} \right], \qquad (S1)$$

where $p_{i,s}$ are the estimated presence probabilities for species i, for the vectors $N = 3$ (*Cx. annulirostris, Cx. sitiens* and *Cx. quinquefasciatus)*, and for the birds $N = 12$ (see Table S1).

The combined enzootic and epidemic risk maps were based on the standard laws of probability, where the probabilistic “or” is given by $P(A \text{ or } B) = P(A) + P(B) - P(A \text{ and } B), \ (S2)$

And the probalistic “and” is given by

$$P(A \text{ and } B) = P(A) \times P(B), \qquad (S3)$$

noting the assumed independence of A and B.
